# DFCNet: Dual-Stage Frequency-Domain Calibration Network for Low-Light Image Enhancement

**DOI:** 10.3390/jimaging11080253

**Published:** 2025-07-28

**Authors:** Hui Zhou, Jun Li, Yaming Mao, Lu Liu, Yiyang Lu

**Affiliations:** School of Automation, Nanjing University of Science and Technology, Nanjing 210094, China; zh123110@njust.edu.cn (H.Z.); 123110223268@njust.edu.cn (Y.M.); 123110223340@njust.edu.cn (L.L.); lyy1217@njust.edu.cn (Y.L.)

**Keywords:** image processing, low-light image enhancement, frequency-domain calibration, feature modulation, unsupervised learning

## Abstract

Imaging technologies are widely used in surveillance, medical diagnostics, and other critical applications. However, under low-light conditions, captured images often suffer from insufficient brightness, blurred details, and excessive noise, degrading quality and hindering downstream tasks. Conventional low-light image enhancement (LLIE) methods not only require annotated data but also often involve heavy models with high computational costs, making them unsuitable for real-time processing. To tackle these challenges, a lightweight and unsupervised LLIE method utilizing a dual-stage frequency-domain calibration network (DFCNet) is proposed. In the first stage, the input image undergoes the preliminary feature modulation (PFM) module to guide the illumination estimation (IE) module in generating a more accurate illumination map. The final enhanced image is obtained by dividing the input by the estimated illumination map. The second stage is used only during training. It applies a frequency-domain residual calibration (FRC) module to the first-stage output, generating a calibration term that is added to the original input to darken dark regions and brighten bright areas. This updated input is then fed back to the PFM and IE modules for parameter optimization. Extensive experiments on benchmark datasets demonstrate that DFCNet achieves superior performance across multiple image quality metrics while delivering visually clearer and more natural results.

## 1. Introduction

Over the past decade, the widespread adoption of imaging technologies in domains including surveillance systems, medical diagnostics, and autonomous driving has established image quality as a critical determinant of overall system performance [1]. In extremely low-light environments, however, image acquisition often results in insufficient brightness, reduced contrast, blurred textures, and significant noise. These degradations compromise both visual appearance and the reliability of downstream vision tasks. For example, in surveillance systems, blurred low-light images may cause target detection and recognition failures [2]. In medical imaging, poor image quality impedes lesion localization and reduces diagnostic accuracy [3]. In autonomous driving scenarios, failure to perceive clear road conditions directly compromises safety [4].

Conventional low-light image enhancement (LLIE) methods, like histogram equalization (HE) and gamma correction, perform basic brightness adjustments but rely exclusively on global gray-level mapping. Such methods lack adaptability to complex illumination distributions and often require manual parameter tuning, rendering them ineffective in diverse, dynamic real-world environments. Over the past few years, approaches grounded in deep learning have shown remarkable progress within LLIE by jointly modeling local details and global illumination features. However, the majority of current approaches are built upon supervised learning with paired low- and normal-light images [5], which involves substantial manual labor for data collection and annotation. Furthermore, many models are computationally intensive and parameter-heavy [6], posing challenges for deployment on resource-constrained devices and limiting their practicality in real-time systems.

To tackle these limitations, a lightweight and unsupervised LLIE framework named dual-stage frequency-domain calibration network (DFCNet) is proposed in this work. The framework employs a dual-stage enhancement architecture: during training, a collaborative dual-stage optimization enhances performance, while at inference, only a single-stage forward pass is needed, effectively balancing accuracy and efficiency. A preliminary feature modulation (PFM) module and an illumination estimation (IE) module are further designed to jointly generate dynamic guidance maps, enabling end-to-end optimization from feature enhancement to illumination estimation. Finally, the frequency-domain residual calibration (FRC) module is applied to compute a calibration term from the stage I output. This calibration term is then fed back into the PFM and IE modules. By using this calibration term to update their parameters, the network acquires the capability to produce superior enhancements in a single inference pass—achieving more natural results and suppressing artifacts without any additional runtime cost.

Extensive experimental results demonstrate that DFCNet outperforms leading supervised and unsupervised baseline methods in enhancement quality, detail preservation, and computational efficiency, thereby showcasing strong practical value and deployment potential. To summarize, the main contributions of this work are outlined as follows:

1. An unsupervised dual-stage framework is proposed to eliminate reliance on paired training data, thus striking a balance between enhancement quality and real-time efficiency.
2. An FRC module is designed to enhance structural consistency while suppressing noise and artifacts by exploiting residual information in the frequency domain.
3. A lightweight PFM module and an IE module are designed to collaboratively facilitate accurate illumination estimation via dynamic feature guidance mechanisms.

The subsequent parts of this work are structured as follows: Section 2 surveys related research in the LLIE domain. Section 3 elaborates on the proposed DFCNet architecture, its submodules, and the loss functions used. Section 4 discusses experimental findings and ablation analyses. Section 5 draws the conclusion.

## 2. Related Work

In recent years, LLIE has attracted significant attention due to its critical role in improving the visibility and quality of images captured under challenging illumination conditions. The main objective of LLIE is to restore clear and natural images from degraded low-light inputs, thereby facilitating subsequent vision tasks such as detection and recognition. Existing LLIE methods can generally be divided into three categories: conventional enhancement methods, supervised learning-based methods, and unsupervised learning-based methods.

### 2.1. Conventional Methods

Conventional LLIE methods typically rely on point-wise gray-level mappings (e.g., HE) and Retinex-based decomposition approaches [7]. Although most gray-level transformations can adjust brightness and contrast, they often need to be combined with other techniques under poor lighting conditions. HE-based methods enhance contrast by dividing the histogram into subbands and equalizing each separately, but they often fail to maintain global illumination consistency. Retinex-based methods improve low-light images by decomposing them into illumination and reflectance components, effectively preserving details [8]. Jobson et al. [9] employed Single-Scale Retinex (SSR) to apply the Retinex theory for intensity consistency and lightness reproduction in gray-level images. A recursive bilateral filter was proposed by Li et al. [10] to significantly expedite the decomposition procedure for enhancing efficiency in LLIE, while Guo et al. [11] proposed the LIME method, which estimates pixel-wise illumination and refines it to reduce computational overhead.

### 2.2. Supervised Deep Learning-Driven Methods

Supervised methods have recently advanced LLIE. These approaches learn low-light to normal-light mappings using large, paired datasets. Lore et al. [12] pioneered LLNet with autoencoders for joint contrast improvement and noise reduction. Lv et al. [13] proposed MBLLEN, which utilizes multi-branch fusion to eliminate noise. Chen et al. [14] proposed SID, exploiting RAW image linearity for extreme LLIE. Lim et al. [15] adopted Laplacian pyramids in DSLR for multi-scale recovery. Building on Retinex theory, RetinexNet by Wei et al. [5] decomposes images into illumination and reflectance for targeted adjustment. Zhang et al. [16] refined this idea in KinD with dedicated decomposition submodules. URetinex was introduced by Wu et al. [17], which integrates denoising and enhancement through adaptive unfolding. Cheng et al. [18] fused light-guided features in LCUN to improve illumination accuracy, while Wang et al. [19] leveraged color priors in LCDPNet for exposure correction. Cai et al. [20] formulated Retinexformer to capture non-local interactions, and Bai et al. [21] accelerated RetinexMamba inference by using state-space modeling. Yao et al. [22] proposed DFFN, a supervised method that fuses spatial and frequency-domain features through dual-domain blocks, adopts a dual-stage design for amplitude and phase optimization, and uses cross-stage fusion to enhance low-light remote sensing images.

Despite their success, supervised methods face limitations. They rely heavily on paired low-/normal-light data, which are rarely available in real-world scenarios. Moreover, synthetic data often poorly reflect real-world conditions, limiting scalability.

### 2.3. Unsupervised Deep Learning-Driven Methods

To overcome the limitations of supervised methods, unsupervised LLIE techniques have emerged as promising alternatives. These methods utilize unpaired datasets, eliminating dependency on ground truth references. Wang et al. [23] proposed a framework called GLADNet, which estimates global illumination via an encoder–decoder architecture to recover detail losses from initial rescaling. EnlightenGAN, introduced by Jiang et al. [6], adopts global and local discriminators with self-supervised regularization, content preservation losses, and attention mechanisms. Zero-DCE, proposed by Guo et al. [24], predicts image-adaptive curves using lightweight networks trained with no-reference losses. RRDNet, introduced by Zhu et al. [25], leverages triple-branch CNNs for joint denoising and restoration. Yang et al. [26] designed DRBN, applying perception-driven linear transformations guided by learned quality assessment networks. RetinexDIP, proposed by Zhao et al. [27], recasts Retinex decomposition as a generative modeling task. Wang et al. [28] created MAGAN, incorporating multi-scale attention for simultaneous enhancement/denoising. Developed by Ma et al. [29], SCI implements a weight-shared and self-calibrated module to learn illumination. Kandula et al. [30] introduced region-adaptive single-input multiple-output generation for user-customized enhancement. CLIP-LIT, presented by Liang et al. [31], leverages CLIP’s semantic priors via prompt learning for illumination adjustment. Jiang et al. [32] proposed UDCN, which decomposes images into illumination and reflectance components, corrects illumination via an adaptive pyramid-based strategy, and removes noise through a dedicated noise removal module. Fei et al. [33] proposed GDP, a generative diffusion prior framework that leverages a pre-trained DDPM model for unsupervised posterior modeling. Luo et al. [34] proposed CRF-inspired plug-and-play frameworks with lightweight curves for self-supervised adaptation. Peng et al. [35] developed DeULLE leveraging luminance masking and illumination-reflectance decoupling under bidirectional cycle supervision.

Although unsupervised methods alleviate the need for paired datasets, many existing architectures remain computationally expensive, making them unsuitable for deployment on resource-constrained devices. Consequently, there is a strong practical imperative to develop efficient, lightweight, unsupervised low-light enhancement approaches that can run in real-time with minimal memory and power overhead. Such a solution would enable broader adoption in applications ranging from on-device surveillance and smartphone photography to battery-powered autonomous systems.

## 3. Proposed Method

### 3.1. Overview of the DFCNet

**Figure 1:**
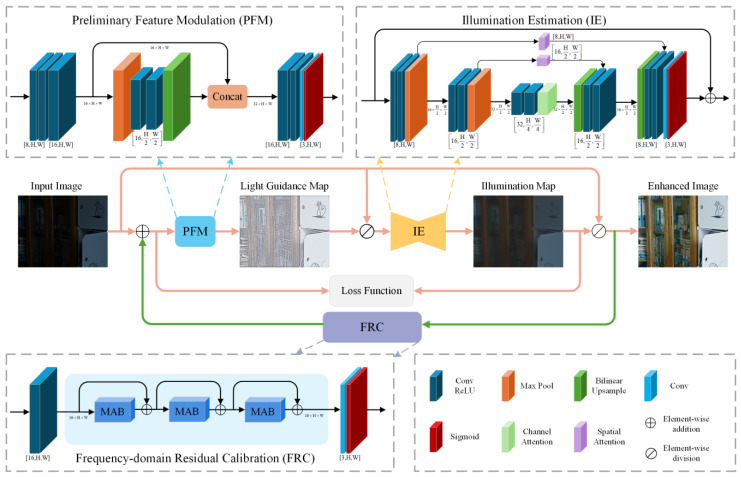
Overview of the proposed DFCNet, including components such as preliminary feature modulation (PFM), illumination estimation (IE), frequency-domain residual calibration (FRC), and the overall workflow. Here, H and W denote the input image’s height and width, respectively. Arrows indicate the flow direction of feature maps between components. The dual-stage process is visually distinguished by colored lines: pink lines indicate the workflow shared by both stages, and green lines mark processes exclusive to the second stage.

The proposed DFCNet in this work employs a dual-stage architecture, depicted in Figure 1. It comprises three core elements: PFM, IE, and FRC, which work together to enhance low-light images efficiently.

During stage I, the input image undergoes PFM processing, where multi-scale convolutional layers extract and fuse features to generate modulated feature maps. These modulated features are then passed to IE, which follows a U-Net-style [36] architecture and incorporates both spatial and channel attention mechanisms to produce an estimated illumination map. The enhanced image is generated via pixel-wise division of the input image by the illumination map, where each pixel in the input is divided by its corresponding pixel in the illumination map.

During stage II, the FRC is designed to further optimize PFM and IE. Specifically, the enhanced image from stage I is passed through FRC to generate a calibration term. This term is added to the original input image, forming the new input for stage II. The pipeline of stage I is then repeated using this updated input. During training, both stages are constrained by separate loss terms to guide the optimization process and improve overall enhancement quality. It is worth noting that during inference, only the first-stage procedure is executed. That is, the input image undergoes processing via PFM and IE to obtain the illumination map, and the final enhanced result is derived via pixel-wise division of the input image by this map. This design ensures high enhancement quality while maintaining inference efficiency.

### 3.2. Preliminary Feature Modulation

Deep feature extraction and adaptive modulation enhance discriminative representations for subsequent illumination estimation and enhancement [37]. To enhance adaptability to diverse low-light scenarios, we designed the PFM with two parallel branches. The main branch conducts progressive feature extraction through multi-layer convolutional layers with ReLU activations to capture fine-grained details and structural information. The downsampling branch enlarges the receptive field using max-pooling, followed by convolutional operations and bilinear upsampling to restore resolution. Features from both branches are fused in a channel-oriented manner for multi-scale fusion, then processed via convolutional layers with ReLU to generate a guidance map. The input image is adaptively modulated through pixel-wise division by this guidance map, resulting in an optimized feature map. This dynamic modulation mechanism adapts subsequent enhancement processes to input-specific feature distributions, significantly enhancing enhancement effectiveness in low-light conditions.

### 3.3. Illumination Estimation

Retinex theory provides an interpretable framework for LLIE and has demonstrated strong generalization capabilities in deep learning applications [38]. It models an input image as the element-wise multiplication of reflectance and illumination: S=R⊙L, where R captures the object’s intrinsic properties, and L represents the illumination affected by external lighting conditions.

To accurately estimate illumination, which is critical for effective reflectance recovery and detail enhancement [39], we designed IE based on the U-Net architecture. The encoder acquires hierarchical features via stacked convolution-ReLU blocks, followed by max pooling to downsample the spatial resolution. A bottleneck layer with channel attention [40] adaptively emphasizes informative channels, capturing global illumination context. The decoder restores spatial resolution through upsampling and integrates shallow details using skip connections with spatial attention [41], enhancing structural awareness. Finally, a Sigmoid-activated convolution layer outputs the estimated illumination map $I\in {\mathbb{R}}^{3\times H\times W}$.

Given the uneven lighting in low-light scenes, a residual enhancement strategy is adopted at the output stage. The input features and the predicted illumination map are fused via weighted addition, correcting estimation errors and reinforcing structural detail for more natural enhancement.

### 3.4. Frequency-Domain Residual Calibration

**Figure 2:**
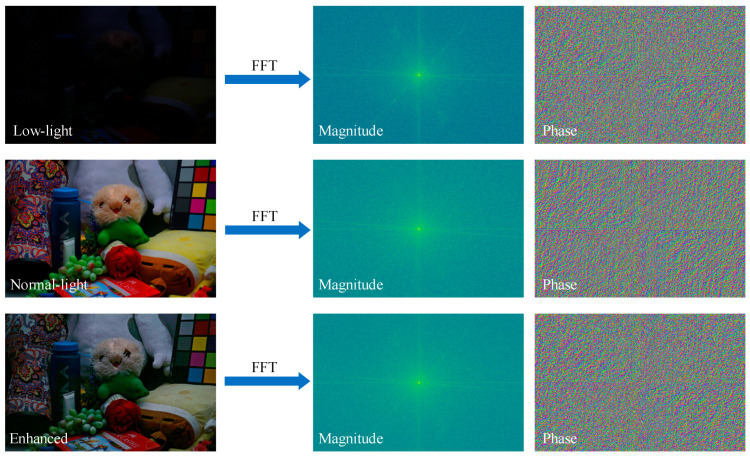
Comparison of magnitudes and phases of low-light, normal-light, and enhanced images.

**Figure 3:**
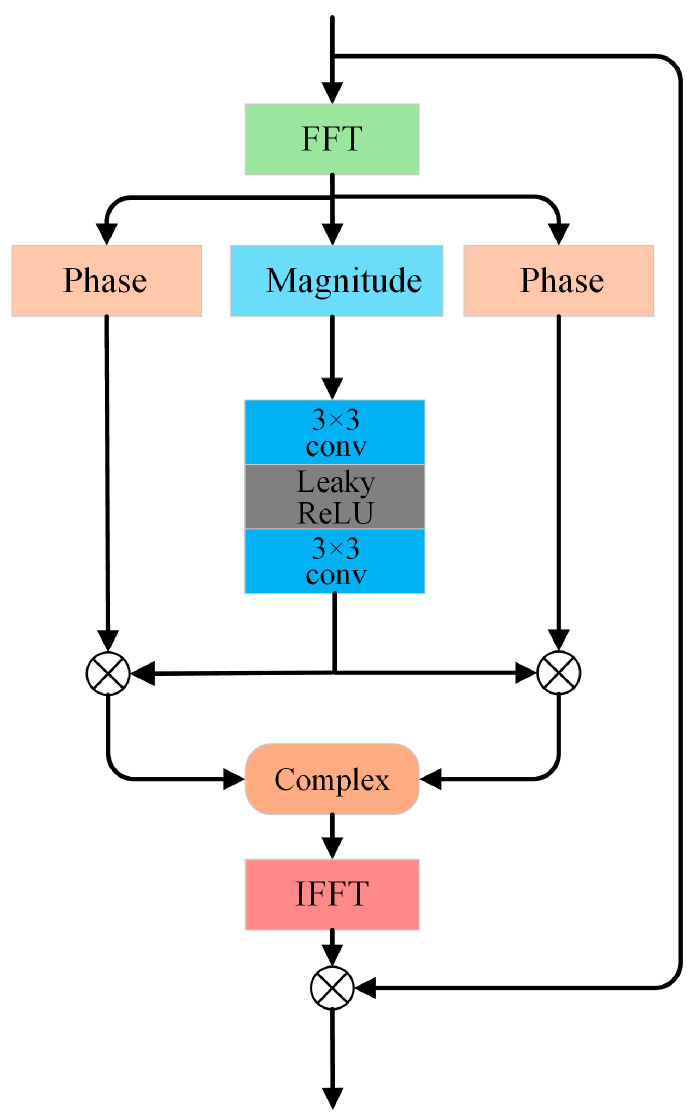
Schematic of the MAB.

Spatial-domain illumination correction methods like the ICN in UDCN [32] have shown effectiveness in adjusting local brightness via adaptive pyramidal processing. Similarly, DFCNet also employs a calibration mechanism, but shifts the focus to the frequency domain. Recent studies have revealed a strong correlation between illumination distributions in low-light conditions and the magnitude characteristics in the frequency domain [42]. As shown in Figure 2, which compares the magnitudes and phases of low-light, normal-light, and enhanced images, explicitly modeling these magnitude features facilitates significant improvements in image brightness and contrast. To improve the modeling of global structures and periodic noise in LLIE networks, FRC is introduced in stage II. In contrast to DFFN [22], which fuses spatial and frequency branches across stages for joint image reconstruction, DFCNet employs a decoupling strategy: its calibration terms yielded from the FRC module serve solely to refine the parameters of the modules in the spatial branch, not to participate directly in image reconstruction. Specifically, FRC mines global magnitude characteristics in the frequency domain to produce a calibration term from the first-stage output. This term is added to the original low-light input, suppressing under-exposed regions and amplifying well-lit areas to yield an updated input. The refined image is then re-fed into the PFM and IE modules, guiding them to learn more robust illumination-aware features while incurring no additional computational overhead at inference. Figure 3 illustrates the magnitude adjustment block (MAB), the central component of the FRC, designed to exploit frequency-domain information for LLIE.

The module takes the reflectance map $R\in {\mathbb{R}}^{3\times H\times W}$ from stage I as input. After shallow encoding, the feature map $F_{in}$ is transformed into the frequency domain via the fast Fourier transform (FFT):

(1) $$F_{freq}\;=F(F_{in}\;)=M\cdot e^{jP}$$

where M represents magnitude and P represents phase. The magnitude component M is then calibrated by a nonlinear unit consisting of channel-expansion convolution, LeakyReLU activation, and channel-reduction convolution.

(2) $$M^{\prime }=W_{2}\;\cdot \mathrm{LeakyReLU}(W_{1}\;\cdot M)$$

This enables adaptive modeling and rescaling of frequency component magnitudes to enhance global structure and noise representation. $M^{\prime }$ is then recombined with P to form the calibrated frequency feature.

(3) $$F_{freq}^{\prime }\;=M^{\prime }\cdot \cos(P)+j\cdot M^{\prime }\cdot \sin(P)=M^{\prime }\cdot e^{jP}$$

The inverse fast Fourier transform (IFFT) maps it back to the spatial domain as $F_{cal}$. For stable information flow and efficient training, $F_{cal}$ is fused with $F_{in}$ via a gated residual connection:

(4) $$F_{out}=F_{in}+F_{cal}⊙F_{in}$$

where ⊙ denotes element-wise multiplication. After iterative MAB processing, final features are processed by a convolutional layer followed by a sigmoid activation function to output the calibration term $C\in {\mathbb{R}}^{3\times H\times W}$, which is afterwards added to the original input image. This resultant sum acts as the input for stage II, enabling further refinement of the global structure and details of the illumination map.

Operating exclusively in stage II, FRC yields calibration terms to aid PFM and IE in learning refined parameters, enabling higher-quality enhancement. Leveraging the strong correlation between frequency-domain magnitude features and low-light illumination distribution, FRC utilizes MAB for efficient frequency-domain modeling and global calibration. This spatial-frequency collaborative modeling strikes a balance between local details and global structures. For clarity, the pipeline of FRC is detailed in Algorithm 1.
**Algorithm 1** Frequency-Domain Residual Calibration (FRC)**Input**Reflectance map $R\in {\mathbb{R}}^{3\times H\times W}$
**Output**Calibration term $C\in {\mathbb{R}}^{3\times H\times W}$
1: Spatial Encoding$F_{in}\;=\mathrm{ReLU}\;(\mathrm{Conv}\;(R))$2: **for** i=0 to k **do**
3:  FFT$F_{freq}\;=F(F_{in}\;)$4:  Magnitude & Phase Separation$M,\;P=\left|F_{freq}\;\right|,\;\mathrm{angle}\;(F_{freq})$5:  Magnitude Calibration$M^{\prime }=\mathrm{Conv}\;(\mathrm{LeakyReLU}\;(\mathrm{Conv}\;(M)))$6:  Frequency Recombination$F_{freq}^{\prime }\;=M^{\prime }\cdot \cos(P)+j\cdot M^{\prime }\cdot \sin(P)$7:  IFFT$F_{cal}\;=F^{-1}(F_{freq}^{\prime }\;)$8:  Residual Fusion with Gating$F_{out}=F_{in}+F_{cal}⊙F_{in}$9:  Update Input$F_{in}\;\;\leftarrow F_{out}$10: **end for**
11: Output Calibration Term$C=\mathrm{Sigmoid}\;(\mathrm{Conv}\;(F_{out}))$

### 3.5. Loss Function

To comprehensively regulate the network, we propose a multi-component loss function to address pixel fidelity, structural smoothness, and noise suppression. Training proceeds in two stages, with losses computed independently at each stage and then summed to form the final optimization objective:

(5) $$L_{\;\mathrm{total}}^{\;t}=\sum_{t=1}^{2}\left(\alpha L_{\;\mathrm{illu}}^{\;t}+\beta L_{\;\mathrm{smooth}}^{\;t}+\gamma L_{\;\mathrm{tv}}^{\;t}\right)$$

where α, β, and γ balance the illumination consistency loss, structural smoothness loss, and total variation loss, respectively, and t indicates the stage. This combined loss supervision ensures that the network not only boosts brightness and contrast but also preserves structural consistency—thereby balancing subjective visual quality with objective evaluation metrics. The total loss is composed of the following three terms.

#### 3.5.1. Illumination Consistency Loss

To ensure that the illumination map at each stage faithfully reflects the input’s overall brightness and structure, we employ a pixel-level loss between the stage’s input and its predicted illumination map:

(6) $$L_{\;\mathrm{illu}}^{\;t}=\sum_{i=1}^{N}∥X_{i}^{t}-I_{i}^{t}{∥}^{2}$$

where $X_{i}^{t}$ denotes the value at pixel i of input to stage t, $I_{i}^{t}$ is the corresponding predicted illumination map, and N represents the total pixel count. Such a loss effectively averts over-enhancement.

#### 3.5.2. Structural Smoothness Loss

Smooth prediction of illumination is important for maintaining the consistency of the spatial structure of illumination maps [43]. To maintain spatial consistency in the illumination map and suppress noise, we adopt a weighted smoothness loss inspired by SCI [29], which helps suppress local noise while preserving edge structures:

(7) $$L_{\;\mathrm{smooth}}^{\;t}=\sum_{i=1}^{N}\sum_{j\in N\left(i\right)}w_{i,j}\cdot \left|I_{i}^{t}-I_{j}^{t}\right|$$

(8) $$w_{i,j}=\exp\left(-\frac{\sum {\left(Y_{i}^{t}-Y_{j}^{t}\right)}^{2}}{2\sigma^{2}}\right)$$

where N(i) denotes the 5 × 5 neighborhood of pixel i, $w_{i,j}$ represents the weight computed from the Y channel (after converting the input image to the standard YUV color space), and σ represents the Gaussian kernel’s standard deviation.

#### 3.5.3. Total Variation Loss

To boost perceptual quality by reducing artifacts and noise, a total variation loss is introduced:

(9) $$L_{\;\mathrm{tv}}^{\;t}=\sum_{i=1}^{N}\sum_{c\in \{r,g,b\}}\left({\left|\nabla_{h}I_{i,c}^{t}\right|}^{2}+{\left|\nabla_{v}I_{i,c}^{t}\right|}^{2}\right)$$

where $I_{i,c}^{t}$ represents the value at pixel i and channel c (RGB) of the predicted illumination map; $\nabla_{h}$ denotes the horizontal operators; and $\nabla_{v}$ denotes vertical gradient operators.

## 4. Experiments

### 4.1. Experiment Settings

The proposed DFCNet was trained on the LOL-v2-real dataset [44], an expanded version of the original LOL dataset [5]. The extended dataset includes 689 low-light/normal-light image pairs for training and 100 pairs for testing, up from the initial 500 pairs. As an unsupervised learning approach is adopted, only the low-light images are utilized during training, without leveraging their paired normal-light counterparts. Experiments were executed on a computer outfitted with a NVIDIA GeForce RTX 4060 Ti GPU (16G) using PyTorch 2.1.0. The training configurations are as follows: a batch size of 8, an initial learning rate of 0.0002, the Adam optimizer (with $\beta_{1}$ = 0.9, $\beta_{2}$ = 0.999, ϵ = 1 × 10^−8^), and a total of 300 training epochs. DFCNet is highly lightweight, with only 0.0529M parameters, even including stage II’s FRC.

### 4.2. Comparative Methods and Evaluation Metrics

For a thorough evaluation of DFCNet’s performance, we compared it against several mainstream LLIE methods, including RetinexNet [5], DSLR [15], RRDNet [25], Zero-DCE [24], EnlightenGAN [6], RetinexDIP [27], LCDPNet [19], SCI [29], CLIP-LIT [31], and GDP [33]. All these methods provide publicly available code with recommended parameters and their corresponding training datasets. They encompass both supervised and unsupervised algorithms, enabling a multifaceted assessment of the strengths and weaknesses of our method.

Evaluation was conducted using both no-reference and full-reference strategies. For perceptual quality, we adopted the no-reference metrics naturalness image quality evaluator (NIQE) [45] and blind/referenceless image spatial quality evaluator (BRISQUE) [46], applied to all test images. For quantitative analysis on paired datasets, we additionally report the full-reference metrics peak-signal-to-noise ratio (PSNR) and structural similarity index measure (SSIM). Recognizing the incompleteness of these metrics in capturing human visual perception, we also include subjective visual comparisons across various scenarios.

The evaluation datasets include LOL [5], and 50 images are randomly sampled from each of MIT [47], ExDark [48], and DarkFace [49] for evaluation. Among them, LOL and MIT are paired datasets that provide low-light images along with their corresponding normal-light references, making them suitable for quantitative evaluation using full-reference metrics. In contrast, ExDark and DarkFace contain only real-world low-light images without reference counterparts, thus better representing practical deployment scenarios and serving as the basis for no-reference evaluation.

### 4.3. Results

The quantitative results comparing our DFCNet to ten other methods on the LOL and MIT datasets (both featuring paired low-/normal-light images) are presented in Table 1. Red and blue are used to mark the best and second-best performers, respectively. On the LOL dataset, DFCNet achieved the second-best performance for both PSNR and NIQE. On the MIT dataset, DFCNet attained the best scores in both PSNR (0.32 higher than the second best) and NIQE (0.14 lower than the second best), while also securing the second-best result for BRISQUE. These results demonstrate DFCNet’s consistent effectiveness across datasets in both reference-based and perceptual quality metrics.

**Figure 4:**
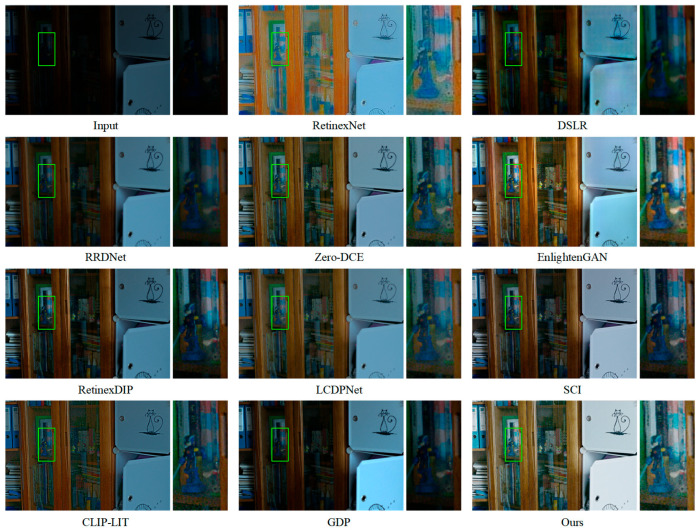
Visual comparison of mainstream methods and DFCNet on the LOL dataset. Right panels show local enlargements of regions marked by green boxes in left panels.

**Figure 5:**
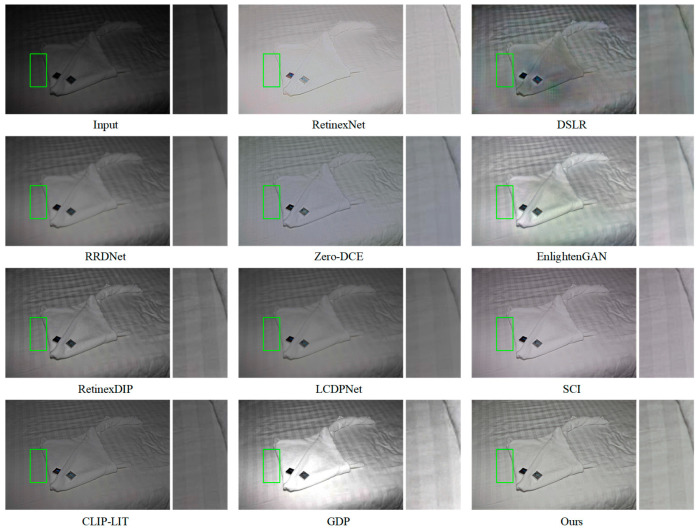
Visual comparison of mainstream methods and DFCNet on the MIT dataset. Right panels show local enlargements of regions marked by green boxes in left panels.

As illustrated in Figure 4, both EnlightenGAN and DFCNet effectively enhanced low-light images. However, DFCNet delivered more significant brightness improvement while better preserving fine details. In contrast, RetinexNet’s results appeared blurry, and DSLR not only suffered from insufficient brightness enhancement but also introduced uneven color artifacts. While other methods provided some enhancement, they generally lacked sufficient brightness improvement, resulting in suboptimal visual results. Figure 5 shows that EnlightenGAN, GDP, and DFCNet all preserved texture information in both overall and zoomed-in views. Nevertheless, EnlightenGAN and DSLR exhibited localized color anomalies, whereas GDP suffered from local overexposure. By comparison, RetinexNet and Zero-DCE produced excessively bright images with low contrast, noticeable distortion, and substantial detail loss. The remaining methods not only failed to effectively enhance image brightness but also struggled with edge preservation between distinct regions, further compromising visual clarity.

The quantitative results comparing our DFCNet to ten other methods on the ExDark and DarkFace datasets are presented in Table 2. As shown, DFCNet achieves the best NIQE score on ExDark (0.05 lower than the second-best method) and attains the second-best BRISQUE score, indicating its superior ability to enhance perceptual quality under real-world low-light conditions. On the DarkFace dataset, DFCNet demonstrates an even more pronounced advantage, achieving the best results for both NIQE and BRISQUE metrics, with scores lower than the second-best method by 0.10 and 1.21, respectively. This consistently strong performance across two challenging datasets, both of which contain diverse and complex low-light scenarios, highlights DFCNet’s robustness and generalization ability in objective image quality evaluation.

**Figure 6:**
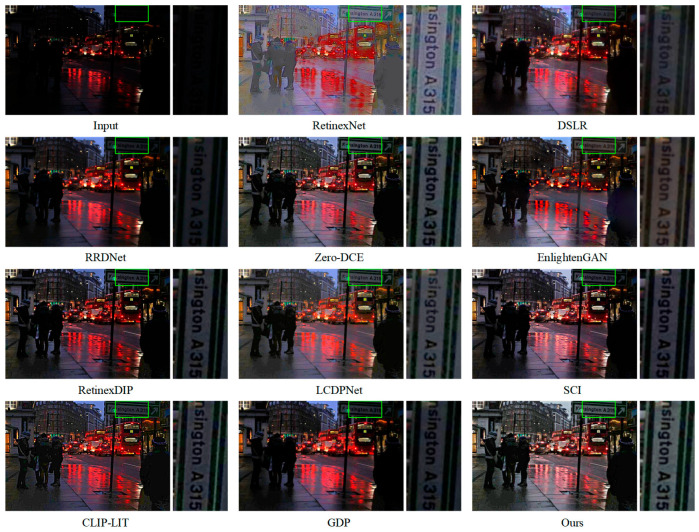
Visual comparison of mainstream methods and DFCNet on the ExDark dataset. Right panels show local enlargements of regions marked by green boxes in left panels.

**Figure 7:**
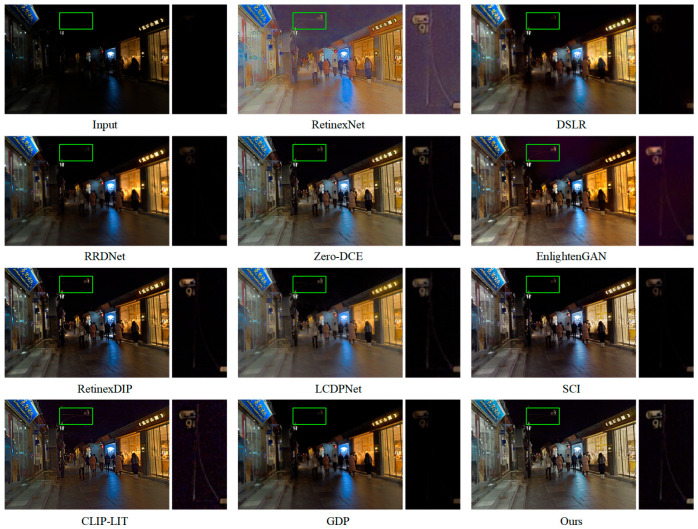
Visual comparison of mainstream methods and DFCNet on the DarkFace dataset. Right panels show local enlargements of regions marked by green boxes in left panels.

Figure 6 visually illustrates the effectiveness of DFCNet. It shows that DFCNet not only restores global brightness but also maintains local contrast by preserving edges between adjacent regions—an essential characteristic for achieving visually pleasing and informative enhancement results in low-light scenarios. In contrast, although RetinexNet manages to improve brightness to some extent, it introduces substantial noise, thereby degrading visual quality and potentially interfering with subsequent image analysis tasks. EnlightenGAN, meanwhile, produces results with a yellowish color cast and degraded fine details, compromising both color fidelity and image realism. Other methods continue to struggle with inadequate brightness enhancement, with RRDNet exhibiting the most deficiencies. Figure 7 further confirms the advantages of our method. DFCNet consistently delivers well-illuminated and visually clear results. By comparison, RetinexNet generates outputs with uneven color distribution, leading to unnatural appearance. In the zoomed-in views, although LCDPNet and CLIP-LIT succeed in brightening local dark areas to some extent, their overall brightness remains inferior to that of DFCNet, indicating limited effectiveness in holistic illumination correction. Notably, while Zero-DCE, EnlightenGAN, SCI, RetinexDIP, and GDP suffer primarily from insufficient global brightness, DSLR and RRDNet not only exhibit inadequate enhancement but also introduce noticeable haze, which further compromises image sharpness and clarity.

In summary, DFCNet offers a balanced trade-off between brightness enhancement, detail preservation, and visual naturalness, providing an effective framework for practical LLIE applications.

### 4.4. Complexity Analysis

**Figure 8:**
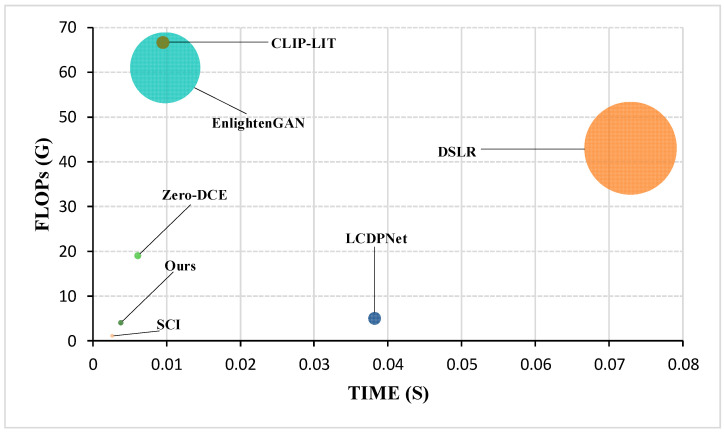
Comparison of computational efficiency across different methods, where the bubble size represents model size (m).

In practical applications of LLIE, the model parameter count and inference speed are critical factors determining the ability to meet real-time requirements. Large models significantly increase memory consumption, while slow inference speed directly impacts user experience or the performance of downstream tasks. To comprehensively assess the overall performance of each method under real-time constraints, we fed low-light images of size 600 × 400 pixels (from the LOL dataset) and 1080 × 720 pixels (from the DarkFace dataset) into their respective models with a batch size of 1, simulating the scenario of processing individual frames in a continuous video stream. It should be noted that RRDNet, RetinexDIP, and GDP do not rely on pre-trained models but instead require hundreds or even thousands of iterative steps to generate enhanced outputs. Due to GDP’s extremely high parameter count and the computational cost of its diffusion process, its FLOPs and inference time are not directly comparable with other models. The results are presented in Table 3, while more intuitive computational efficiency comparisons are visualized in Figure 8. Our DFCNet exhibits exceptional competitiveness on both key metrics: it boasts a remarkably low parameter count of merely 0.0428M and requires only 0.0038 s to enhance a single image on the 600 × 400 LOL dataset (with 0.0051 s on the 1080 × 720 DarkFace dataset). This extremely low parameter count makes it the second lightest model in this comparison, significantly lower than EnlightenGAN (8.637M), while achieving comparable enhancement quality. In terms of computational cost (floating point operations, FLOPs), DFCNet shows favorable efficiency across resolutions, with 4.0263G on LOL and 13.0454G on DarkFace, ranking competitively. However, the inference time of the second-ranked method in parameter compactness, RetinexDIP, is exceedingly slow (reaching 16.2311 s per image on LOL and 16.8708 s on DarkFace), severely limiting its practical deployment. Although the parameter count and per-image inference time of DFCNet are higher than those of the top-ranked SCI, DFCNet achieves superior enhancement results. In summary, DFCNet’s performance demonstrates its ability to handle real-time LLIE tasks efficiently, striking an optimal balance between model compactness and computational efficiency across diverse low-light image resolutions.

### 4.5. Ablation Study

**Figure 9:**
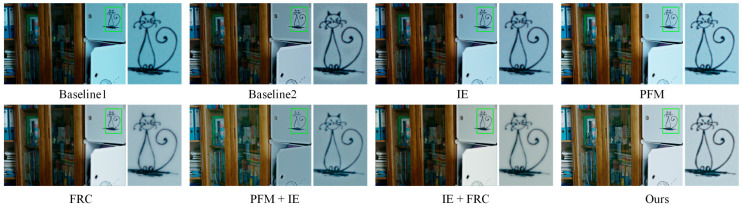
Visualization results of the ablation experiments on PFM, IE, and FRC conducted on the LOL dataset. Right panels show local enlargements of regions marked by green boxes in left panels.

The effectiveness of the proposed PFM, IE, and FRC modules within DFCNet was evaluated through eight ablation experiments conducted on the LOL dataset. Performance was assessed using PSNR, SSIM, NIQE, and BRISQUE metrics. Detailed experimental configurations and corresponding results are presented in Table 4. To further illustrate the impact of each module on visual quality, visual comparisons of enhancement results are provided in Figure 9, where the right panel displays zoomed-in details of key regions. Baseline1 replaces the IE module with standard convolutional layers (employing ReLU activations) of equivalent depth for illumination estimation. Baseline2, in contrast, substitutes the FRC module with a multi-head spatial-frequency convolution block. Visual enhancement results, illustrated in Figure 9, demonstrate DFCNet’s superior performance. Compared to the cascaded convolutions in Baseline1, our IE module achieves more accurate illumination prediction, yielding enhanced images with higher overall brightness. Unlike the multi-head convolution block in Baseline2, whose redundant heads amplify noise within the sparse frequency spectrum of static images (leading to color fringing and blurred edges), our single-branch FRC effectively suppresses these artifacts, preserving clean and color-consistent boundaries. Guided by the PFM module, the IE module focuses on capturing extremely dark regions, enabling better illumination prediction while preserving image details during the enhancement process. The inclusion of the FRC module effectively corrects color casts in the enhanced images, producing more natural and visually pleasing results. The synergistic integration of all three modules achieves optimal performance across all evaluation metrics, highlighting their complementary roles in LLIE.

## 5. Conclusions

To address the issue of degraded image quality under low-light imaging conditions, an innovative unsupervised LLIE method named DFCNet is proposed. DFCNet effectively overcomes the limitations of traditional supervised approaches, particularly their inability to meet real-time processing requirements. It achieves this through a dual-stage workflow. In the first stage, DFCNet utilizes PFM to modulate low-light images, guiding the network toward more accurate illumination estimation. The reflectance map, representing the enhanced output, is then obtained by dividing each pixel of the input image by the corresponding value in the estimated illumination map. In the second stage, FRC is introduced to further optimize network performance, significantly improving the quality of the enhanced images. DFCNet not only improves the usability of low-light images but also demonstrates strong potential in providing more reliable visual data in practical applications such as security surveillance and autonomous driving. Future work will explore deploying DFCNet in broader real-world scenarios to further extend its applicability and impact.

## Figures and Tables

**Table 1:** Qualitative results of mainstream methods and DFCNet on LOL and MIT datasets. Red and blue numbers are used to mark the best and second-best performers, respectively. ↑ Represents the higher the value is better, whereas ↓ means the lower the value is better.

Methods	Venue	LOL				MIT			
		PSNR ↑	SSIM ↑	NIQE ↓	BRISQUE ↓	PSNR ↑	SSIM ↑	NIQE ↓	BRISQUE ↓
RetinexNet	BMVC’18	16.77 ± 2.38	0.564 ± 0.077	8.39 ± 0.53	35.43 ± 7.69	14.29 ± 1.89	0.698 ± 0.087	4.58 ± 0.92	29.24 ± 8.59
DSLR	TMM’20	14.95 ± 4.16	0.600 ± 0.131	8.18 ± 0.48	33.78 ± 8.75	18.17 ± 3.88	0.769 ± 0.047	3.83 ± 0.79	32.66 ± 10.05
RRDNet	ICME’20	10.70 ± 3.65	0.436 ± 0.148	7.82 ± 0.91	29.32 ± 8.27	16.16 ± 3.69	0.664 ± 0.068	4.08 ± 0.88	30.20 ± 10.22
Zero-DCE	CVPR’20	14.86 ± 4.27	0.562 ± 0.124	7.85 ± 1.03	24.98 ± 8.10	16.07 ± 1.95	0.738 ± 0.061	3.65 ± 0.76	24.31 ± 8.52
EnlightenGAN	TIP’21	17.48 ± 3.53	0.651 ± 0.110	5.40 ± 0.69	23.77 ± 5.89	17.95 ± 2.44	0.791 ± 0.060	3.19 ± 0.70	28.14 ± 9.49
RetinexDIP	TCSVT’21	11.68 ± 3.86	0.487 ± 0.136	7.57 ± 1.08	22.55 ± 6.89	18.41 ± 3.11	0.836 ± 0.049	3.23 ± 0.67	22.86 ± 8.83
LCDPNet	ECCV’22	14.51 ± 4.94	0.575 ± 0.098	7.44 ± 0.79	22.63 ± 7.37	17.07 ± 2.52	0.770 ± 0.065	3.24 ± 0.74	25.75 ± 10.03
SCI	CVPR’22	14.81 ± 4.08	0.548 ± 0.137	7.81 ± 1.06	27.32 ± 6.60	18.06 ± 3.10	0.815 ± 0.063	3.55 ± 0.79	25.55 ± 8.26
CLIP-LIT	ICCV’23	12.39 ± 3.62	0.493 ± 0.121	7.90 ± 0.97	28.10 ± 8.08	17.19 ± 2.94	0.773 ± 0.066	3.38 ± 0.76	23.90 ± 9.27
GDP	CVPR’23	15.90 ± 2.38	0.542 ± 0.077	7.95 ± 0.73	30.14 ± 7.69	18.26 ± 2.89	0.799 ± 0.087	3.22 ± 0.92	24.76 ± 8.59
Ours	-	17.04 ± 3.32	0.592 ± 0.107	7.12 ± 0.79	24.06 ± 7.68	18.73 ± 2.26	0.787 ± 0.069	3.05 ± 0.71	23.11 ± 8.48

**Table 2:** Qualitative results of mainstream methods and DFCNet on ExDark and DarkFace datasets. Red and blue numbers are used to mark the best and second-best performers, respectively. ↓ means the lower the value is better.

Methods	Venue	ExDark		DarkFace	
		NIQE ↓	BRISQUE ↓	NIQE ↓	BRISQUE ↓
RetinexNet	BMVC’18	4.44 ± 0.68	31.64 ± 9.05	4.42 ± 0.22	34.67 ± 2.83
DSLR	TMM’20	3.90 ± 0.64	28.65 ± 8.55	3.74 ± 0.38	34.90 ± 4.17
RRDNet	ICME’20	3.65 ± 0.99	20.77 ± 7.45	3.97 ± 0.44	30.34 ± 3.59
Zero-DCE	CVPR’20	3.03 ± 0.58	27.72 ± 7.04	3.50 ± 0.45	28.18 ± 5.16
EnlightenGAN	TIP’21	3.12 ± 0.63	19.61 ± 6.81	3.26 ± 0.33	26.61 ± 3.22
RetinexDIP	TCSVT’21	3.32 ± 0.63	25.76 ± 6.81	3.58 ± 0.43	24.50 ± 4.27
LCDPNet	ECCV’22	3.27 ± 0.81	20.22 ± 7.54	3.23 ± 0.29	28.62 ± 4.92
SCI	CVPR’22	2.97 ± 0.66	21.65 ± 7.15	3.36 ± 0.44	24.71 ± 3.26
CLIP-LIT	ICCV’23	3.16 ± 0.66	23.65 ± 8.42	3.73 ± 0.48	32.46 ± 6.35
GDP	CVPR’23	3.21 ± 0.68	25.45 ± 7.05	3.68 ± 0.22	29.16 ± 3.83
Ours	-	2.92 ± 0.57	20.03 ± 6.98	3.13 ± 0.37	23.29 ± 2.40

**Table 3:** Model sizes, FLOPs, and single-image inference times of different models under real-time constraints (GPU-based). Red and blue numbers are used to mark the best and second-best performers, respectively.

Methods	Venue	SIZE(M)	LOL (600 × 400)		DarkFace (1080 × 720)	
			FLOPs (G)	TIME (S)	FLOPs (G)	TIME (S)
RetinexNet	BMVC’18	0.8383	136.0151	0.1131	441.4513	0.5533
DSLR	TMM’20	14.9313	43.0319	0.0729	141.0071	0.0451
RRDNet	ICME’20	0.1282	30.6500	90.4702	99.3212	272.6918
Zero-DCE	CVPR’20	0.0794	19.0081	0.0061	61.5859	0.0076
EnlightenGAN	TIP’21	8.6370	61.0261	0.0098	196.5670	0.1028
RetinexDIP	TCSVT’21	0.7072	3.4134	16.2311	11.2549	16.8708
LCDPNet	ECCV’22	0.2818	5.0068	0.0382	15.9825	0.0286
SCI	CVPR’22	0.0003	0.1282	0.0025	0.4152	0.0033
CLIP-LIT	ICCV’23	0.2788	66.6701	0.0095	216.0111	0.0165
Ours	-	0.0428	4.0263	0.0038	13.0454	0.0051

**Table 4:** Qualitative results of ablation experiments on the LOL dataset. √ Indicates the module is used. Red and blue numbers are used to mark the best and second-best performers, respectively. ↑ Represents the higher the value is better, whereas ↓ means the lower the value is better.

Baseline	PFM	IE	FRC	PSNR ↑	SSIM ↑	NIQE ↓	BRISQUE ↓
Baseline1				10.049	0.381	8.556	31.650
Baseline2				12.414	0.460	7.374	26.561
		√		12.348	0.456	8.139	28.632
	√			13.496	0.467	7.935	28.247
			√	12.919	0.475	7.442	26.354
	√	√		15.985	0.574	7.644	27.468
		√	√	15.455	0.569	7.309	25.919
	√	√	√	17.039	0.592	7.115	24.060

## Data Availability

The original contributions presented in this study are included in the article. Further inquiries can be directed to the corresponding author.
